# Isotretinoin-Related Eye Dryness in Acne Patients in Qassim, Saudi Arabia

**DOI:** 10.7759/cureus.49904

**Published:** 2023-12-04

**Authors:** Yasmeen A Alfouzan, Rana A Al-Hammad, Ferial A Alkhuzayem, Raghad F Alkhudair, Mzoun A Alotaibi, Abdullah N Alajaji, Ahmed A Al-Muhaylib

**Affiliations:** 1 College of Medicine, Qassim University, Buraydah, SAU; 2 Department of Dermatology, Qassim University Medical City (QUMC), Buraydah, SAU; 3 Department of Ophthalmology, Unaizah College of Medicine and Medical Sciences, Qassim University, Unaizah, SAU

**Keywords:** qassim, saudi arabia, acne patients, eye dryness, isotretinoin

## Abstract

Background

Isotretinoin is a commonly prescribed agent mainly used to treat acne vulgaris. Since its Food and Drug Administration (FDA) approval, the subject of its associations with various medical conditions has become a growing interest in many investigational studies.

Methodology

A retrospective study was conducted using the validated Standardized Patient Evaluation of Eye Dryness (SPEED) questionnaire. All statistical calculations were performed using the Statistical Package for the Social Sciences (SPSS) version 27.0.1 (IBM SPSS Statistics, Armonk, NY).

Results

The research study involved 107 participants, predominantly Saudi nationals (98.1%), with an average age of 22 years. Regarding the total cumulative dose (TCD), 40.2% had less than 50 mg/kg, 33.6% had between 50 and 100 mg/kg, and 15.9% had more than 100 mg/kg. The majority (73.8%) did not stop using isotretinoin. The average Ocular Surface Disease Index (OSDI) score, indicating ocular surface disease symptoms, was 26.78. There was no significant association between gender, dose/duration of isotretinoin, TCD, and age, and OSDI scores. However, participants with severe OSDI scores were more likely to have worsened pre-existing eye symptoms compared to those with non-severe OSDI scores (35.1% versus 11.4%, p = 0.011).

Conclusions

This study contributes to the understanding of the impact of isotretinoin usage on ocular surface health. While no significant associations were found between gender, dose, duration, TCD, and age, and OSDI scores, participants with pre-existing eye symptoms were more likely to experience worsened symptoms during isotretinoin treatment.

## Introduction

Isotretinoin is a commonly prescribed oral retinoid primarily used for the treatment of severe acne and various other skin disorders [1]. As reported in a systematic review done by Vos et al. (2012) of the Global Burden of Disease study, acne vulgaris is the eighth most prevalent disorder around the world [2]. Consequently, with the global rise in the use of isotretinoin particularly among those between the ages of 12 and 24 [3], it is of great importance for physicians to be aware of its safety profile and to recognize the issues and adverse side effects associated with its use [4].

Ocular adverse effects (AEs) are common with isotretinoin use, most of which occur as a result of alterations to the corneal surface or abnormalities of lacrimation that lead to inadequate moisture and dry eyes [5]. In the study that evaluated ocular AEs related to isotretinoin use, Fraunfelder et al. (2001) categorized ocular AEs using the World Health Organization classification system for Causality Assessment of Suspected Adverse Reactions [6].

The potential implications of eye dryness on patients' quality of life are particularly significant, and they demand our attention [7]. Dry eye symptoms, if left unaddressed, can substantially affect daily activities, including reading, using electronic devices, and driving, thereby diminishing the overall well-being of individuals under treatment [8]. By exploring the subjective experiences and discomfort faced by our patients, we hope to gain a comprehensive understanding of the multifaceted implications of eye dryness [9].

Our dermatology clinics at Qassim University have treated several patients with acne via isotretinoin treatment, and a number of these patients have reported ocular manifestations and severe eye dryness. Due to the scarcity of studies on this subject in our population, our study aims to identify the prevalence of eye dryness that manifests in these patients and assess the frequency and severity of this isotretinoin-related eye dryness.

## Materials and methods

In this study, a community-based retrospective approach was employed. The study duration spanned one year, commencing in November 2021 and concluding in November 2022, during which approximately 200 participants were selected for the sample. In the process of participant selection, specific inclusion and exclusion criteria were established. Inclusion criteria encompassed patients of any gender who had undergone oral isotretinoin treatment for acne for a minimum of four weeks and were actively taking oral isotretinoin. Patients ineligible for this study included those undergoing any other systemic or ocular treatments, individuals wearing contact lenses, or those with a history of dry eye, keratitis, allergic ocular disease, ocular surface disease, glaucoma, active and chronic uveitis, or previous ocular surgery or injury prior to their isotretinoin treatment. For data collection, the researchers administered the online questionnaire upon gaining ethical approval. This questionnaire was the validated Standardized Patient Evaluation of Eye Dryness (SPEED) questionnaire [10]. In cases where participants expressed a preference for a verbal questionnaire session, this alternative method was also considered.

Simple descriptive statistics of the sociodemographic characteristics and other categorical variables in the form of frequencies and percentages were calculated and tabulated. For continuous variables, means and standard deviations (SDs) were reported as measures of central tendency and dispersion, respectively. The Ocular Surface Disease Index (OSDI) score of the participants was calculated by the standard procedure of multiplying the total score on the questionnaire by 100 divided by four times the number of questions answered. Thus, the total OSDI scores ranged from 0 to 100. An OSDI score of greater than 33 was considered a severe disease as per the established criteria.

To find the association of total score with sociodemographic characteristics, either the independent samples t-test or one-way analysis of variance (ANOVA) were applied and interpreted as the statistical methods of choice for comparison of mean OSDI scores between the various groups. In addition, Fisher's exact test was applied for the comparison of categorical variables where needed. Significance was established at a p-value of 0.05 or less with a 95% confidence interval. All statistical calculations were performed using the Statistical Package for the Social Sciences (SPSS) version 27.0.1 (IBM SPSS Statistics, Armonk, NY).

## Results

The study included 107 participants satisfying the inclusion criteria, predominantly Saudi nationals (98.1%). Females represented 61.7% of the participants. The average age was 22 years (SD = 3.77 years). The average height was 162.99 cm (SD = 23.79 cm), and the average weight was 65.13 kg (SD = 18.27 kg) (Table 1).

**Table 1:** Sociodemographic Characteristics of the Participants

Characteristics		Number	%
Total		107	100%
Nationality	Saudi	105	98.1%
	Other	2	1.9%
Gender	Male	41	38.3%
	Female	66	61.7%
Age (years)		22	3.77
Height (cm)		162.99	23.79
Weight (kg)		65.13	18.27

In terms of the duration of isotretinoin usage, the majority of participants (44.9%) used it for 4-6 months, while 38.3% used it for 1-3 months. A small percentage (0.9%) used it for less than a month, and 7.5% used it for more than six months. Additionally, 8.4% of participants were off isotretinoin recently. Regarding the daily dosage, 61.7% of the participants received a dose of 40 mg/day, making it the most common dosage. A smaller percentage (30.8%) received a dose of 20 mg/day, while only 7.5% received a dose of 30 mg/day. The average dose per kilogram of body weight per day was 0.53 (SD = 0.17). The mean duration of isotretinoin usage was 121.31 days (SD = 53.01 days). Participants were further classified based on the daily dose and duration. Among them, 39.3% received a moderate dose, defined as >30 mg/day for 1-3 months or <20 mg/day for 4-6 months or more than six months. Meanwhile, 38.3% received a high dose, defined as >30 mg/day for ≥4 months. Only 12.1% received a low dose, defined as <20 mg/day for 1-3 months. In terms of the total cumulative dose (TCD) classification, 40.2% of the participants had a TCD of less than 50 mg/kg, 33.6% had a TCD between 50 and 100 mg/kg, and 15.9% had a TCD exceeding 100 mg/kg. Furthermore, 10.3% of the participants had not used isotretinoin for more than two months. Regarding the stoppage of isotretinoin use, the majority of participants (73.8%) had not stopped using it. Among those who did stop, 15.9% ceased usage less than two months ago, while 10.3% stopped more than two months ago. The average OSDI score, which indicates ocular surface disease symptoms, was 26.78 (SD = 19.97) (Table 2).

**Table 2:** Dosage and Duration of Isotretinoin Usage and OSDI Score OSDI: Ocular Surface Disease Index, M: mean, SD: standard deviation, TCD: total cumulative dose

Dose and duration		Number	%	M	SD
For how long have you been using isotretinoin?	Less than a month	1	0.9%		
	1-3 months	41	38.3%		
	4-6 months	48	44.9%		
	More than 6 months	8	7.5%		
	Not currently using it	9	8.4%		
Dose (mg/day)	20	33	30.8%		
	30	8	7.5%		
	40	66	61.7%		
Dose (mg/kg/day)				0.53	0.17
Days used				121.31	53.01
TCD				64.14	34.78
Classification based on daily dose and duration	Low dose (<20 mg/day for <1-3 months)	13	12.1%		
	Moderate dose (>30 mg/day for 1-3 months or <20 mg/day for 4-6 months or >6 months)	42	39.3%		
	High dose (>30 mg for 4-6 months or >6 months)	41	38.3%		
	Stopped for >2 months after low dose	2	1.9%		
	Stopped for >2 months after medium/high dose	9	8.4%		
Classification based on TCD	<50 mg/kg	43	40.2%		
	50-100 mg/kg	36	33.6%		
	>100 mg/kg	17	15.9%		
	Not used for more than 2 months	11	10.3%		
Stopped using isotretinoin since when	Not stopped	79	73.8%		
	Since less than 2 months	17	15.9%		
	Since more than 2 months	11	10.3%		
OSDI score				26.78	19.97

Prior to the treatment course, the majority (69.2%) did not experience any eye symptoms, while 11.2% reported pre-existing symptoms that worsened and 19.6% experienced new symptoms. During the treatment course, 54.2% of the participants used lubricant eye drops when needed, while 38.3% used them daily or most of the time. Only a small percentage (7.5%) did not use lubricant eye drops at all. For patients who completed their treatment course, 5.6% reported that their symptoms disappeared completely, while 11.2% experienced a reduction in symptom severity. Only 0.9% still suffered from symptoms after treatment. The majority of participants (95.3%) had not undergone laser eye surgery before starting isotretinoin. Among those who did have laser eye surgery, it occurred more than one year prior to starting isotretinoin (Table 3).

**Table 3:** Baseline Clinical Characteristics of the Participants

		Number	%
Suffering from these eye symptoms prior to treatment course	No, not at all	74	69.2%
	Yes	12	11.2%
	Yes, but they became worse	21	19.6%
Used lubricant eye drops during the treatment course	No, not at all	8	7.5
	Yes, daily/most of the time	41	38.3
	Yes, when needed	58	54.2
For patients who have ended their course, did symptoms continue after stopping the treatment?	No, they have disappeared	6	5.6%
	Yes, but they are less severe now	12	11.2%
	Yes, I am still suffering from them	1	0.9%
Had laser eye surgery before the isotretinoin treatment course	No	102	95.3%
	Yes	5	4.7%
If (yes), when was it in relation to isotretinoin treatment course?	More than 1 year prior to starting isotretinoin	5	4.7%

**Figure 1:**
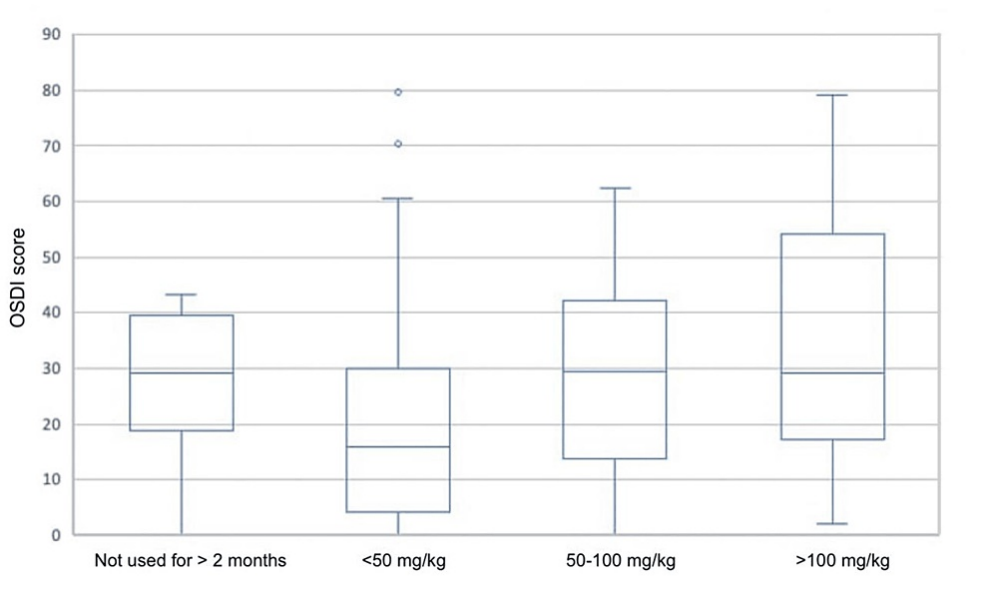
Association of Total Cumulative Dose of Isotretinoin With OSDI Score OSDI: Ocular Surface Disease Index

The mean OSDI score for males was 22.53 (SD = 19.32), while for females, it was 29.42 (SD = 20.06). Although a slight difference was observed, the p-value (0.083) indicated a non-significant association between gender and OSDI scores. When examining the classification based on daily dose and duration, participants who received a low dose (<20 mg/day for <1-3 months) had a mean OSDI score of 18.98 (SD = 17.41), while those on a moderate dose (>30 mg/day for 1-3 months or <20 mg/day for 4-6 months or more than six months) had a mean OSDI score of 26.11 (SD = 21.25). Participants on a high dose (>30 mg/day for 4-6 months or more than six months) had the highest mean OSDI score of 29.66 (SD = 20.74). The p-value for this association was 0.267, indicating no significant relationship between dose/duration and OSDI scores. For the TCD categories, participants with a TCD of <50 mg/kg had a mean OSDI score of 21.17 (SD = 21.07), while those with a TCD between 50 and 100 mg/kg had a mean OSDI score of 29.44 (SD = 16.92). Participants with a TCD exceeding 100 mg/kg had the highest mean OSDI score of 34.68 (SD = 23.95). The p-value for this association was 0.079, suggesting a non-significant relationship between TCD and OSDI scores. Lastly, the correlation analysis between age and OSDI scores showed a Pearson correlation coefficient of 0.116, indicating a weak positive correlation. However, the p-value (0.235) indicated no significant association between age and OSDI scores (Table 4, Figure 1).

**Table 4:** Association of Gender and Dose/Duration of Isotretinoin With OSDI Scores A: one-way analysis of variance, t: independent samples t-test OSDI: Ocular Surface Disease Index, M: mean, SD: standard deviation, TCD: total cumulative dose

		OSDI score		p-value^A,t^
		M	SD	
Gender	Male	22.53	19.32	0.083
	Female	29.42	20.06	
Classification based on daily dose and duration	Low dose (<20 mg/day for <1-3 months)	18.98	17.41	0.267
	Moderate dose (>30 mg/day for 1-3 months or <20 mg/day for 4-6 months or >6 months)	26.11	21.25	
	High dose (>30 mg for 4-6 months or >6 months)	29.66	20.74	
	Stopped for >2 months after low dose	8.33	11.79	
	Stopped for >2 months after medium/high dose	32.08	9.29	
TCD categories	<50 mg/kg	21.17	21.07	0.079
	50-100 mg/kg	29.44	16.92	
	>100 mg/kg	34.68	23.95	
	Not used for more than 2 months	27.77	13.24	

**Figure 2:**
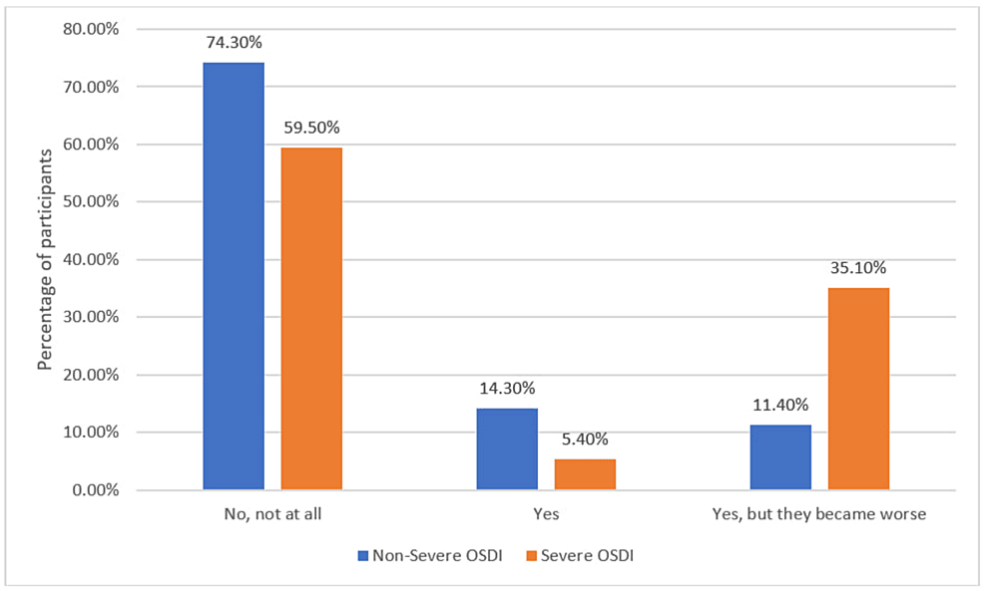
Percentage of Participants With Severe OSDI (33-100)/Non-severe OSDI (>33) Suffering From Eye Symptoms Prior to Treatment OSDI: Ocular Surface Disease Index

Looking at the percentage of participants with non-severe OSDI (scores below 33) and severe OSDI (scores ranging from 33 to 100) who reported suffering from eye symptoms prior to the treatment course, among participants with non-severe OSDI scores, 74.3% did not have any eye symptoms prior to treatment, while 14.3% reported pre-existing symptoms, and 11.4% mentioned that their symptoms had worsened. For participants with severe OSDI scores, 59.5% reported no eye symptoms prior to treatment, while 5.4% had pre-existing symptoms, and 35.1% experienced a worsening of their symptoms (Figure 2).

## Discussion

Our study aimed to investigate the association between isotretinoin usage and ocular surface disease symptoms, as measured by the Ocular Surface Disease Index (OSDI) scores. The study included 107 participants who met the inclusion criteria, primarily consisting of Saudi nationals, with a majority of female participants.

Demographic characteristics of the study participants indicated that the majority were Saudi nationals. Females constituted a significant proportion of the sample, comprising 61.7% of the participants. The average age of the participants was 22 years, with a relatively narrow standard deviation, suggesting a relatively homogeneous age group. The average height and weight of the participants were within typical ranges.

Regarding the dosage and duration of isotretinoin usage, the majority of participants used the medication for 4-6 months (44.9%) or 1-3 months (38.3%). The most common daily dosage was 40 mg/day (61.7%). These findings suggest that the participants in this study generally adhere to the recommended treatment duration and dosage [11]. However, it is worth noting that a small percentage (0.9%) used isotretinoin for less than a month, which could be due to various factors such as discontinuation due to adverse effects or incomplete treatment courses. When classifying participants based on daily dose and duration, it was found that the majority received a moderate dose (39.3%), followed by a high dose (38.3%), and a low dose (12.1%).

Our study examined the ocular effects of isotretinoin treatment in a sample of participants. We categorized the participants based on their total cumulative dose (TCD) of isotretinoin and evaluated the impact of treatment on ocular symptoms and the frequency of isotretinoin use cessation. In terms of TCD classification, our findings revealed that 40.2% of the participants had a TCD of less than 50 mg/kg, 33.6% had a TCD between 50 and 100 mg/kg, and 15.9% had a TCD exceeding 100 mg/kg. It provides insights into the distribution of isotretinoin dosages within our sample. These results are consistent with previous studies conducted by AlMasoudi et al. (2022) [5] and Leyden et al. (2014) [12], which reported similar distributions of TCD categories in their respective study populations.

Regarding the cessation of isotretinoin use, the majority of participants (73.8%) had not stopped using it. This suggests that most individuals in our sample continued their isotretinoin treatment throughout the study period. However, among those who did stop using isotretinoin, 15.9% ceased usage less than two months ago, while 10.3% stopped more than two months ago. These findings align with the study, which reported similar proportions of participants who discontinued isotretinoin usage within different timeframes [13,14].

In terms of ocular surface disease symptoms, we utilized the Ocular Surface Disease Index (OSDI) score as a measure of symptom severity. The average OSDI score in our study was 26.78 (SD = 19.97), indicating a moderate level of ocular surface disease symptoms among participants. These results are consistent with the findings of Zakrzewska et al. (2023) [15], who also reported moderate OSDI scores in their study investigating the ocular effects of isotretinoin treatment.

Before initiating the treatment course, the majority of participants (69.2%) did not experience any eye symptoms. However, 11.2% reported pre-existing symptoms that worsened, while 19.6% experienced new symptoms. It highlights the potential ocular effects of isotretinoin, including both the exacerbation of pre-existing symptoms and the development of new symptoms. It underscores the importance of monitoring and addressing ocular changes during isotretinoin treatment to ensure the well-being and visual health of the patients. This supports the findings of Jarab et al. (2022) [16], who found a comparable percentage of participants experiencing worsened or new eye symptoms prior to isotretinoin treatment.

During the treatment course, the use of lubricant eye drops was common among participants, with 54.2% using them when needed and 38.3% using them daily or most of the time. Only a small percentage (7.5%) did not use lubricant eye drops at all. The high percentage of participants using lubricant eye drops indicates the need for proactive management of ocular symptoms to ensure patient comfort and minimize potential ocular side effects during isotretinoin treatment. These results are in line with the study conducted by Basheikh et al. (2020) [17], where they observed a similar pattern of lubricant eye drop usage during isotretinoin treatment.

For patients who completed their treatment course, 5.6% reported complete disappearance of eye symptoms, while 11.2% experienced a reduction in symptom severity. A mere 0.9% of participants still suffered from symptoms after treatment. These findings are consistent with the study by Bagatin et al. (2020) [18], who reported a comparable proportion of participants achieving symptom resolution or reduction after completing isotretinoin treatment.

Regarding the history of laser eye surgery, the majority of participants (95.3%) had not undergone the procedure before starting isotretinoin. Among the small number of participants who had laser eye surgery, it occurred more than one year prior to starting isotretinoin. It implies that any ocular effects observed during the study were less likely to be influenced by recent surgical interventions.

The association between OSDI scores and various factors, such as age, gender, dose, and duration, was examined. The results revealed a non-significant association between gender and OSDI scores, indicating that gender may not be a significant predictor of ocular surface disease symptoms. Similarly, no significant association was found between the dose and duration of isotretinoin usage and OSDI scores, suggesting that higher doses or longer durations may not necessarily lead to more severe symptoms. These findings suggest that other factors might contribute to the development of ocular surface disease symptoms during isotretinoin treatment.

The TCD classification also showed a non-significant relationship with OSDI scores, indicating that the cumulative dose of isotretinoin might not be a significant factor in the development of ocular surface disease symptoms. However, it is important to note that a higher TCD exceeding 100 mg/kg was associated with higher mean OSDI scores, suggesting a potential trend that warrants further investigation with larger sample sizes.

The correlation analysis between age and OSDI scores revealed a weak positive correlation, but the p-value indicated no significant association. This finding suggests that age might not be a prominent factor influencing ocular surface disease symptoms in this sample.

The results revealed that a larger proportion of participants with severe OSDI scores experienced a worsening of their pre-existing eye symptoms compared to those with non-severe OSDI scores. These findings suggest that the severity of OSDI scores may be influenced by the presence and worsening of pre-existing eye symptoms. Our study's results are consistent with previous research that has examined the relationship between pre-existing symptoms and OSDI severity. For instance, Asghari et al. (2022) [19] conducted a similar study and reported that patients with severe OSDI scores were more likely to have pre-existing symptoms compared to those with non-severe scores. This supports the notion that pre-existing symptoms play a role in the severity of OSDI scores, and our findings further highlight the impact of symptom worsening.

Furthermore, our study also adds to the existing literature by demonstrating that a significantly larger proportion of participants with severe OSDI scores experienced a worsening of their pre-existing eye symptoms after isotretinoin treatment compared to those with non-severe OSDI scores (35.1% versus 11.4%). This finding suggests that isotretinoin may have an exacerbating effect on pre-existing eye symptoms, particularly in individuals with more severe OSDI scores [9,20].

It is important to note that the mechanism underlying the exacerbation of pre-existing eye symptoms other than dryness by isotretinoin treatment remains unclear and requires further investigation. However, it is possible that isotretinoin's known side effects, such as dryness and irritation of all body mucosal membranes, could contribute to the worsening of other pre-existing eye symptoms in susceptible individuals [21]. Future studies should explore the specific pathways through which isotretinoin influences ocular symptoms and consider potential interventions to mitigate these effects.

It is important to acknowledge the limitations of this study. The relatively small sample size and the predominantly limited area may have limited the generalizability of the findings. Future studies with larger and more diverse samples are warranted to further explore the relationship between isotretinoin usage and ocular surface disease symptoms. Additionally, longitudinal studies tracking participants before, during, and after isotretinoin treatment could provide valuable insights into the temporal changes in ocular surface health.

## Conclusions

This study contributes to the understanding of the impact of isotretinoin usage on ocular surface health. While no significant associations were found between gender, dose, duration, TCD, and age with OSDI scores, participants with pre-existing eye symptoms were more likely to experience worsened symptoms during isotretinoin treatment. These findings emphasize the importance of individual assessment and monitoring of ocular surface health during isotretinoin therapy. Hence, we recommend using it with caution and proper monitoring for those with pre-existing eye problems. Further research is needed to validate and expand upon these findings, with a focus on larger sample sizes and diverse populations.
